# Characterization of the Gut Microbiota of the Antarctic Heart Urchin (Spatangoida) *Abatus agassizii*

**DOI:** 10.3389/fmicb.2020.00308

**Published:** 2020-02-28

**Authors:** Guillaume Schwob, Léa Cabrol, Elie Poulin, Julieta Orlando

**Affiliations:** ^1^Laboratorio de Ecología Molecular, Instituto de Ecología y Biodiversidad, Facultad de Ciencias, Universidad de Chile, Santiago, Chile; ^2^Laboratorio de Ecología Microbiana, Departamento de Ciencias Ecológicas, Facultad de Ciencias, Universidad de Chile, Santiago, Chile; ^3^Aix Marseille University, Univ Toulon, CNRS, IRD, Mediterranean Institute of Oceanography (MIO) UM 110, Marseille, France

**Keywords:** heart sea urchin, deposit-feeder, *Abatus agassizii*, gut microbiota, core-microbiota, keystone, West Antarctic Peninsula, Antarctica

## Abstract

*Abatus agassizii* is an irregular sea urchin species that inhabits shallow waters of South Georgia and South Shetlands Islands. As a deposit-feeder, *A. agassizii* nutrition relies on the ingestion of the surrounding sediment in which it lives barely burrowed. Despite the low complexity of its feeding habit, it harbors a long and twice-looped digestive tract suggesting that it may host a complex bacterial community. Here, we characterized the gut microbiota of specimens from two *A. agassizii* populations at the south of the King George Island in the West Antarctic Peninsula. Using a metabarcoding approach targeting the 16S rRNA gene, we characterized the *Abatus* microbiota composition and putative functional capacity, evaluating its differentiation among the gut content and the gut tissue in comparison with the external sediment. Additionally, we aimed to define a core gut microbiota between *A. agassizii* populations to identify potential keystone bacterial taxa. Our results show that the diversity and the composition of the microbiota, at both genetic and predicted functional levels, were mostly driven by the sample type, and to a lesser extent by the population location. Specific bacterial taxa, belonging mostly to *Planctomycetacia* and *Spirochaetia*, were differently enriched in the gut content and the gut tissue, respectively. Predictive functional profiles revealed higher abundance of specific pathways, as the sulfur cycle in the gut content and the amino acid metabolism, in the gut tissue. Further, the definition of a core microbiota allowed to obtain evidence of specific localization of bacterial taxa and the identification of potential keystone taxa assigned to the *Desulfobacula* and *Spirochaeta* genera as potentially host selected. The ecological relevance of these keystone taxa in the host metabolism is discussed.

## Introduction

Sea urchins (Echinodermata: Echinoid, Leske 1778) constitute one of the most abundant and ecologically important components of marine benthic ecosystems (Steneck, 2013). They represent over 900 species and have colonized all seabed around the world (Littlewood and Smith, 1995). Most of them, called “regular” species harbor a radial body symmetry, an epifaunal lifestyle in energy-rich ecosystems and are mainly deep-grazing herbivores (De Ridder et al., 1982). Contrastingly, the “irregular” sea urchins (Irregularia Latreille, 1825) comprise an infra-class of the Echinoid, favored in oligotrophic environments and characterized by bilateral symmetry and a range of infaunal behaviors (Ziegler, 2014). This divergence in lifestyle is associated with a marked change in nourishment strategy. As a matter of fact, the irregular sea urchins are major deposit-feeders, feeding on detritus particles by the ingestion of surface sediment (De Ridder et al., 1987). Due to the substantial mixing and resuspension of the soft sediments through their feeding and movements (Hollertz and Duchêne, 2001; Lohrer et al., 2005), the irregular sea urchins from the order Spatangoida, commonly called heart urchins, are considered among the most active bioturbators of marine ecosystems (Queirós et al., 2013). These spatangoid urchins are the largest group of irregular echinoids with more than 200 living species and are particularly represented in the Antarctic continent (Ghiold, 1989; Thompson and Riddle, 2005).

Among the spatangoid urchins, the genus *Abatus* Troschel (family Schizasteridae) regroups 11 nominative species specifically distributed in distinct sub-Antarctic and Antarctic provinces. They are usually encountered burrowed few centimeters below the surface of muddy to sandy sediments, from very shallow waters down to 1000 m depth (Poulin and Feral, 1995; David et al., 2005). They are characterized as “poor-dispersers” owing to their direct development (i.e., no larval phase) and the release of the offspring from four dorsal marsupials in the immediate vicinity of the females (Poulin and Feral, 1995; David et al., 2005; Gil et al., 2009; Ledoux et al., 2012). Due to its abundance, wide (sub)Antarctic distribution and easy availability, the *Abatus* genus has been used as a model in numerous studies in the Southern Ocean biology areas such as embryology (Schatt and Feral, 1996), reproductive biology (Maturana et al., 2017), biogeography (Gil et al., 2009; Díaz et al., 2012) and ecology (Magniez, 1983; Lawrence et al., 1984; Palma et al., 2007; Guillaumot et al., 2018). Given its low dispersal and recolonization capacities, and its marked sensitivity to the modifications of seawater properties, *Abatus* is considered as a highly vulnerable species (Ledoux et al., 2012; Saucède et al., 2017; Guillaumot et al., 2018). As recently suggested for regular sea urchins (Brothers et al., 2018), the *Abatus*-associated microbiota might play a role in its host tolerance toward environmental changes.

The first evidence of the microbiota contribution to sea urchin metabolism was provided by Lasker and Giese (1954). The authors demonstrated that the bacteria from the gut of the herbivore sea urchin *Strongylocentrotus purpuratus* were able to digest the alga *Iridophycus flaccidum* within a week completely. Since then, there is a growing body of data about the diversity and the composition of the gut microbiota from grazing, wood-feeding and scraping regular echinoids (Becker et al., 2009; Zhang et al., 2014; Hakim et al., 2016; Hakim et al., 2019; Yao et al., 2019). Moreover, the implication of regular sea urchin microbiota has been documented in various aspects of the host life such as its digestion (Sawabe et al., 1995; Thorsen et al., 2003; Lawrence et al., 2013), its immunity (Becker et al., 2009) and the nutrient transfer (Sauchyn and Scheibling, 2009). On the other hand, gut microbiota characterization of irregular sea urchins is scarcer and focused on a single species: *Echinocardium cordatum* from the Atlantic coast of Western Europe (Da Silva et al., 2006). As a result of its infaunal life and its feeding on detritus particles, the *E. cordatum* digestive tract has been described as mostly anoxic and was characterized by the predominance of fermentative bacteria able to metabolize refractile and sulphated carbohydrates in the ingested sediment (Thorsen, 1998; Da Silva et al., 2006). The degradation of this organic matter leads to high concentration of sulfates that are in turn reduced by sulfate-reducing bacteria, such as Desulfonema. Constrastingly, Thorsen (1998) showed that the immediate vicinity of *E. cordatum* gut was locally more oxygenated, particularly at intestinal nodules coated with microbial mats that host filamentous sulfur-oxidizing bacteria from the *Thiotrix* genus (Brigmon and De Ridder, 1998). These bacteria would be able to re-oxidize the sulfide produced by sulfate-reducing bacteria, providing a detoxifying effect to the host (De Ridder and Brigmon, 2003; Da Silva et al., 2006).

The microbial communities associated with echinoderms have been rarely investigated in Antarctica (Giudice et al., 2019). To the best of our knowledge, the microbiota of a single regular sea urchin species (i.e., *Sterechinus neumayeri*) has been explored in the Antarctic continent through a culture-dependent approach, highlighting the antibiotic and metal resistance capacity of isolated bacteria from the coelomic fluid of *S. neumayeri* collected in Fildes Peninsula, King George Island (González-Aravena et al., 2016). Despite its status of a model genus and its ecological importance in the Antarctic and Subantarctic benthic ecosystems, there is no description of the gut microbiota of the deposit-feeder *Abatus. Abatus agassizii* (Mortensen, 1951) is an endemic irregular sea urchin species distributed in sporadic and dense populations, primarily between 1 and 10 m depth in sheltered bays from the Fildes Bay of King George Island, South Shetland Islands, Antarctica (Palma et al., 2007; Díaz et al., 2012). Notwithstanding the low complexity of its feeding behavior (i.e., sediment filtration), it harbors a long (i.e., about three times the length of the individual) and twice-looped digestive tract, characterized by slow food transit (i.e., 72–97 h) (Thompson and Riddle, 2005), and consisting in several regions – buccal cavity, anterior esophagus, posterior esophagus, anterior stomach, posterior stomach, intestine and rectum – all filled with sediment (Ziegler, 2014). Thus the structure and the physiology of *A. agassizii* gut are strong evidence supporting the presence of a unique, resident and beneficial gut microbiota that may contribute to the host nutrition.

In the present study, we provide a comprehensive description of *A. agassizii* gut microbiota through a 16S rRNA metabarcoding approach focusing on three types of samples: the gut content, the gut tissue and the surrounding sediment. Our goals were (i) to evaluate the differentiation of the microbiota in terms of diversity, community composition and putative functional capacity among the sample types, (ii) to determine the existence of a core microbiota in *A. agassizii* individuals from distinct populations, (iii) to investigate the co-occurrence patterns among the core bacterial taxa to highlight potential keystone within hosts microbiota, and (iv) to analyze the contribution of neutral processes (i.e., ecological drift and passive dispersal) to *Abatus* microbiota assembly.

## Materials and Methods

### Sample Collection and Preparation

Adult *Abatus agassizii* sea urchins (*n* = 14) were sampled in January 2019 from two populations less than 2 km apart, in a sandy habitat at 7 m depth off the coast of the Ardley Island (62°12′32.4″S 58°55′44.399″O) (referred to as “Ardley” site), and at 2 m depth off the coast of the Antarctic China Base “Great Wall” (62°13′7.644°S 58°57′28.295”O) (referred to as “China” site), in the Fildes Peninsula, King George Island, South Shetland Islands (Antarctica). Once collected, sea urchin specimens were immediately stored in a tank at 4°C containing *in situ* seawater and sediment. Between 1–12 h after sampling, sea urchins were measured and dissected to collect the whole gut content (i.e., ingested sediment filling the digestive tract). The gut tissue (i.e., whole gut except the caecum) was excised and gently rinsed with nuclease-free sterile water (Winkler, Santiago, Chile). Superficial sediment samples from the immediate vicinity of the *A. agassizii* were also collected as the food source of the sea urchins (*n* = 3 and *n* = *5* in Ardley and China sites, respectively). A total of 36 samples – 8 external sediments, 14 gut contents and 14 gut tissues – were collected and kept frozen at −20°C for molecular analyses.

### DNA Extraction, 16S rRNA Amplification and Sequencing

Gut tissue samples were homogenized using a mortar and pestle in aseptic conditions. Genomic DNA was extracted from 0.35 g of defrosted samples from the gut content and the external sediments, and from the totality of the homogenized gut tissue samples, using the DNeasy^®^ PowerSoil^®^ Kit (Qiagen, Hilden, Germany) according to the manufacturer’s protocol. An amplicon library of the V4-V5 hypervariable region of the 16S rRNA gene was created using the primers 515F 5′-GTGYCAGCMGCCGCGGTA-3′ and 926R 5′-CCCCGYCAATTCMTTTRAGT-3′ (Parada et al., 2016), tagged with Illumina adaptor sequences supplied by the sequencing company. PCR amplification was performed with a mix containing 5 μL template DNA (40–200 ng), 0.5 mM of each primer, 25 μL of Phusion Hot Start II High-Fidelity PCR Master Mix (Thermo Fisher Scientific, Vilnius, Lithuania) and nuclease-free water (Thermo Fisher Scientific, Vilnius, Lithuania, *q.s.* 50 μL). The reaction conditions were an initial denaturation of 3 min at 98°C, 32 two-step cycles of 10 s at 98°C and 15 s at 72°C, and a final extension step of 5 min at 72°C. Quantity and quality of the PCR products were checked on an electrophoresis gel. Amplicons were fused with Illumina barcodes and sequencing was completed using the paired-end sequencing technology (2 × 250 bp) with the chemistry V2 on Illumina MiSeq platform at Genotoul GIS facility (Toulouse, France).

### Metabarcoding Data Processing and Analysis

Reverse and forward reads generated from the 36 samples were processed using the open-source software Mothur (v.1.42.3) (Schloss et al., 2009). Reads were paired and trimmed according to their length and their content in homopolymers and ambiguous bases. The criteria were one single error in the primer sequence, a maximum of 8 homopolymers and 0 ambiguous bases. Short (<370 nt) and long (>380 nt) reads were discarded. Chimeras were removed with Uchime implemented in Mothur (Edgar et al., 2011). Cleaned sequences were aligned using the *kmer* searching function in SILVA alignments database (release 132) and were assigned in a taxonomic classification (SILVA database) (Quast et al., 2013). Any affiliations to chloroplasts, mitochondria or Eukarya were removed from the dataset. Processed sequences were then clustered into operational taxonomic units (OTU) at 97% identity. To eliminate erroneous sequences and assignments, OTUs with relative abundance <0.005% were discarded as recommended in Bokulich et al. (2013). Samples were rarefied to the number of sequences present in the smallest sample dataset (6100 sequences). The Fastq files generated in this study have been deposited at the National Center for Biotechnology Information (NCBI)^1^ under the project accession number PRJNA590493 (submission ID: SUB6567135).

### Bacterial Community Diversity and Composition

Rarefaction curves were calculated from the non-rarefied OTU table, with samples pooled according to their type and site of provenance, using the *rarefaction.single* function implemented in Mothur, and were then visualized in R with the *ggplot* package. The 95% confidence intervals of observed richness were calculated directly in Mothur with 5000 iterations (randomization). Richness, diversity indices and Bray–Curtis dissimilarity matrix were calculated from the rarefied OTU table using the *vegan* package in R (Oksanen et al., 2011). Richness and diversity indices were compared among sample types using the non-parametric Kruskal–Wallis test followed by a *post hoc* Dunn’s test in R with a correction of *p-value* using the Holm’s method (significance threshold *p* < 0.05) (Dunn, 1961) to determine which groups were different. Taxonomic assignments were pooled at the class level, filtered at 0.05% abundance and comparisons among sample types were performed using the same test and parameters previously mentioned. Additionally, a Venn diagram based on OTU presence/absence was computed from the rarefied OTU table using the *VennDiagram* R package. Bray–Curtis matrix was used to perform non-metric multidimensional scaling (NMDS) with the *metaMDS* function, associated with the *envfit* function to identify the discriminant OTUs most correlated with the samples’ ordination (vegan package). Permanova (permutational multivariate analysis of variance) using both *adonis* and *pairwise.adonis* functions, was applied to investigate the contribution of site and sample type contribution in the samples’ clustering. These functions are implemented in the *vegan* and *pairwiseAdonis* R packages, respectively, Oksanen et al. (2011), Martinez Arbizu (2017). Pairwise comparisons were performed using the R package *limma*, with moderated *t*-tests on log-transformed OTU relative abundance and corrected for multiple hypothesis testing (Smyth, 2005; Bulgarelli et al., 2012) to identify the differentially abundant OTUs in each of the three sample types. Focusing on the significantly enriched OTUs in each sample type compared to the two others (*p*-value < 0.05), a heatmap and a ternary plot were produced [*vegan* package and personal R script adapted from Bulgarelli et al. (2012)].

### Functional Predictions

Functional predictions of the bacterial communities from the gut content and the gut tissue were determined through the Phylogenetic Investigation of Communities by Reconstruction of Unobserved States (PICRUSt v2.0.0) as described in Douglas et al. (2019). The PICRUSt pipeline was run using the representative sequences of the OTUs from the rarefied OTU table using the *get.oturep* function in Mothur. Hidden state predictions were performed through the *hsp.py* script by the Maximum Parsimony method for 16S rRNA gene copy number, Enzyme Commission (EC) numbers and Kyoto Encyclopedia of Genes and Genomes Orthology (KO) abundances, along with the calculation of the Nearest Sequenced Taxon Index (NSTI). Any OTU-representative sequence with an NSTI value higher than two was classified as uncharacterized phyla and was discarded for further analysis. Then, the copy numbers of gene families were predicted for each OTU with the *metagenome_pipeline.py* script, providing the OTU abundances per sample normalized by the predicted 16S rRNA gene copy numbers per OTU. Finally, pathway-level abundances were predicted with *pathway_pipeline.py* function assigning EC numbers to MetaCyc reactions and KO abundances in Kyoto Encyclopedia of Genes and Genomes (KEGG) pathways. Potential functional profiles were analyzed using the STAMP (v2.1.3) software (Parks et al., 2014). Comparisons between sample type community features were achieved through Welch’s *t*-test, *p*-values were adjusted with the Benjamini–Hochberg FDR multiple-test correction, and comparisons with *p*-value < 0.05 were considered significant.

### Core Microbiota, Co-occurrence Networks and Neutral Model

A core microbiota was defined for each gut-related sample type – gut content and gut tissue – as the fraction of the community common to both Ardley and China sites. Shared OTUs were defined as OTUs prevalent in 75% of the samples from the same type in both sites. Co-occurrence analysis was performed on the gut core microbiota of each site through the Co-occurrence Network software (CoNet, v1.1.1) (Faust and Raes, 2016), following the approach described in Hakim et al. (2019). Briefly, OTUs with a cumulative sum of relative abundance per sample lower than 100 (i.e., <15% sequence relative abundance) were discarded from the analysis. Co-occurrence scores were computed using two correlation (Pearson, Spearman), two dissimilarity (Kullback–Leibler, Bray–Curtis) and one similarity (mutual information) measures detailed in Faust and Raes (2016). The 100 most positive and most negative edges, which represent the top 100 co-presence and co-exclusion, respectively, were selected for the randomization step. Multi-edges scores were randomized by bootstrapping at 100 iterations, unstable edges were filtered out, and *p*-values were merged using the Brown method and Benjamini–Hochberg method for multiple test correction (Brown, 1975; Choma et al., 2016). The significance threshold for corrected *p*-values of edges was set at *p* < 0.05. Co-occurrence networks were visualized and edited in Cytoscape v3.7.1 (Shannon et al., 2003) using the Compound Spring Embedder (CoSE) layout. Topological parameters (i.e., average network centralization, characteristic path length, average number of neighbors, network density and heterogeneity) were determined with the plugin NetworkAnalyzer (v2.7) implemented in Cytoscape. For each taxon of both networks, three-node parameters, the number of edges, the closeness centrality, and the betweenness centrality, were plotted using the *ggplot* R package (Wickham, 2006). Hub taxa were manually identified based on their high centrality and connectivity within each network compared with the other taxa (Berry and Widder, 2014; Agler et al., 2016).

The potential contribution of neutral processes (i.e., stochastic factors such as ecological drift and passive dispersion) in microbiota assemblies from gut content, gut tissue and the combination of both was assessed by the neutral community model proposed by Sloan et al. (2006), applying the R code of Burns et al. (2016). The migration rate estimator (*m*) was used to evaluate if the observed frequencies and mean relative abundances of OTUs fit the neutral model. The fit performance to the sample type was assessed with a generalized R-squared method (Burns et al., 2016). The Akaike Information Criterion (AIC) was used to assess the fit between neutral model and a binomial model, the latter representing a random sampling from the metacommunity without drift or dispersal limitations. Well-predicted OTUs supposedly driven by stochastic factors were comprised within the 95% confidence limits, while deviated OTUs that fell above or below the 95% confidence interval were more likely influenced by deterministic factors (i.e., microbial interactions, host selection, among others).

## Results

### Sequencing and Data Processing

A total of 1,298,790 reverse and forward raw reads was generated from the 36 samples. After the sequencing processing, 851,938 cleaned sequences were clustered into 113,088 unique OTUs at 97% identity. The OTU table was then condensed into 1015 unique OTUs and 652,123 sequences applying the <0.005% relative abundance threshold. Read numbers per sample varied from 6100 to 32,000. An equivalent mean of reads per sample was obtained for Ardley and China sites (19,083 versus 18,253 on average, respectively). The rarefaction curves of the samples pooled according to the sample type and site nearly reached saturation suggesting an overall good sequencing coverage (Supplementary Figure S1).

### Changes in Alpha Diversity Along the Host Gradient

All alpha diversity analysis performed (observed richness, Shannon and Simpson indices) led to the same gradient of microbial diversity among the external sediments (highest diversity), the gut content (intermediate diversity) and the gut tissue (lowest diversity) on China site (Dunn test, *p*-values < 0.05) and pooled sites dataset (Dunn test, *p*-values < 0.02) (Table 1 and Supplementary Table S1). The samples from Ardley site tend to harbor the same pattern of diversity (gut content < gut tissue and external sediment < gut tissue, Dunn test, *p*-values < 0.02), at the exception of the external sediment and the gut content samples that did not showed any significant difference on richness, Shannon and Simpson indices (Dunn tests, *p*-values = 0.076) (Supplementary Table S1).

**TABLE 1:** Alpha diversity of Abatus agassizii gut and external sediment microbiota.

**Site**	**Sample types**	**Replicates**	**Richness**	**Shannon index**	**Simpson index**
Ardley	External sediment	3	547.0 ± 3.5^a^	5.18 ± 0.06^a^	0.99 ± 0.002^a^
	Gut content	7	390.7 ± 21.1^a^	4.70 ± 0.04^a^	0.98 ± 0.002^a^
	Gut tissue	7	104.1 ± 15.5^b^	2.03 ± 0.23^b^	0.72 ± 0.080^b^
China	External sediment	5	559.2 ± 6.1^a^	5.24 ± 0.03^a^	0.98 ± 0.001^a^
	Gut content	7	372.3 ± 26.0^b^	4.17 ± 0.06^b^	0.94 ± 0.005^b^
	Gut tissue	7	120.6 ± 17.0^C^	2.39 ± 0.20^c^	0.79 ± 0.040^c^
Sites pooled	External sediment	8	554.6 ± 4.4^a^	5.22 ± 0.03^a^	0.98 ± 0.001^a^
	Gut content	14	381.5 ± 15.7^b^	4.43 ± 0.08^b^	0.96 ± 0.006^b^
	Gut tissue	14	112.4 ± 10.9^C^	2.21 ± 0.15^c^	0.76 ± 0.042^c^

*Average values ± standard errors are presented. Letters represent the significance of sample type comparisons based on Dunn tests (*p* < 0.05).*

### Gut and External Sediment Bacterial Community Composition

**FIGURE 1:**
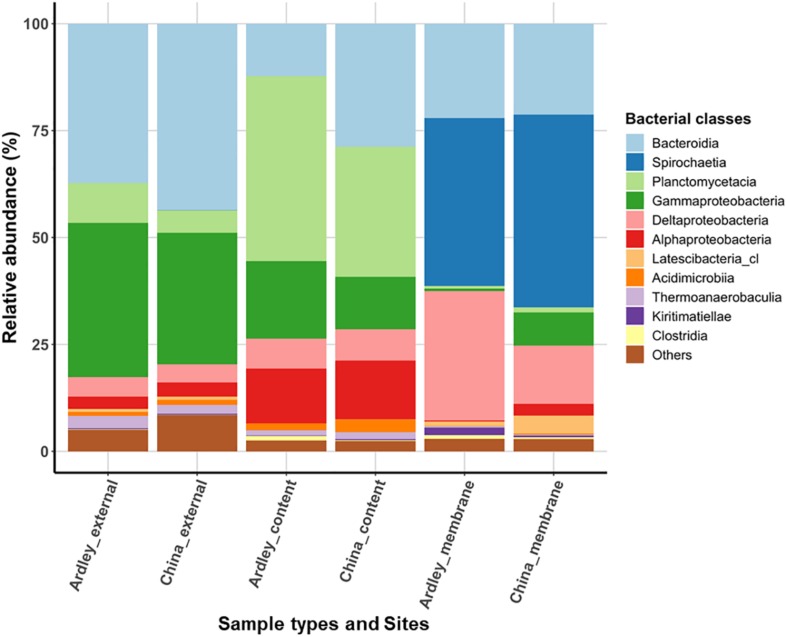
Relative abundance of bacterial classes in *Abatus agassizii* gut and external sediment microbiota in Ardley and China sampling sites. Bacterial classes were filtered at >0.5% relative abundance threshold and the average of the replicate abundances are presented for each condition (i.e., for each combination of sites and sample types). Each color represents a distinct bacterial class.

Applying a filter at >0.5% of class relative abundance, we identified 11 bacterial classes (Figure 1) and 113 genera (data not shown) among *A. agassizii* gut microbiota and external sediment samples. External sediment samples of Ardley and China sites were characterized by co-dominance of *Bacteroidia* (Ardley 37.2% and China 43.6%) and *Gammaproteobacteria* (Ardley 36.1% and China 30.7%), and in a lesser extent of *Planctomycetacia* (Ardley 9.3% and China 5.3%) (Figure 1). When comparing bacterial class abundances among sample types at intra- and inter-site levels, the same pattern was essentially conserved between them, despite minor but significant variations in the abundance of some bacterial classes between the two sites (Supplementary Table S2). The same classes were maintained between the external sediment and the gut content, but their relative abundances varied. The gut content samples were characterized by two major significant enrichments, in *Planctomycetacia* becoming the dominant class (43.3 and 30.4% of the total community for Ardley and China, respectively), and in *Alphaproteobacteria* (12.8 and 13.8% of the total community for Ardley and China, respectively), as well as an impoverishment in *Gammaproteobacteria* (18.1 and 12.3% of the total community for Ardley and China, respectively), and *Bacteroidia* classes (12.2% of the total community and significant for Ardley only), with respect to the external sediment samples.

The most striking taxonomic shifts were observed in the gut tissue samples, mainly dominated by the class *Spirochaetia* (39.3 and 45.1% of the total community for Ardley and China, respectively), that was barely undetected in the two other sample types (<0.03 ± 0.03% total community, on average), and followed by *Deltaproteobacteria* (30.3 and 13.6% of the total community for Ardley and China, respectively). The relative abundances of *Gammaproteobacteria* (0.6 and 7.8% of the total community for Ardley and China, respectively), and *Planctomycetacia* (0.6 and 1.1% of the total community for Ardley and China, respectively), also decreased in the gut tissue in comparison with the gut content samples (Figure 1 and Supplementary Table S2). Unlike the external sediment and the gut content samples, there was no significant differences between the gut tissue samples of Ardley and China sites, mainly because of the high variability between replicates (Supplementary Table S2).

### Beta-Diversity of Bacterial Community Among Sites and Sample Types

**FIGURE 2:**
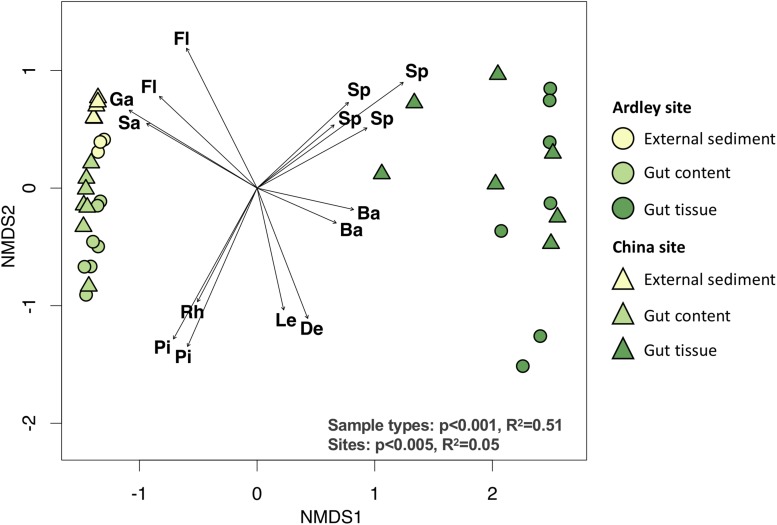
Non-metric multidimensional scaling ordination of the bacterial community composition of the *Abatus agassizii* gut and external sediment microbiota. Colors are assigned to the different sample types. Circles and triangles represent samples from Ardley and China sites, respectively. The arrows represent the variations of the 15 discriminant OTUs most significantly correlated with community ordination (envfit, *p* < 0.05). The letters indicate the taxonomic identification at the family level of the discriminant OTUs (Ba: *Bacteroidetes BD2-2*, De: *Desulfobacteraceae*, Fl: *Flavobacteriaceae*, Ga: *Gammaproteobacteria* unclassified, Le: *Lentimicrobiaceae*, Pi: *Pirullulaceae*, Rh: *Rhodobacteraceae*, Sa: *Sandaracinaceae*, Sp: *Spirochaetaceae*). Effect of the sample type and the site was tested using permutational multivariate analysis of variance (Adonis test) on Bray–Curtis distance matrix computed from the OTU table (details provided in Supplementary Table S2).

We compared *A. agassizii* bacterial community composition in the different sites and sample types through non-metric multidimensional scaling (NMDS) computed from Bray–Curtis distances. In both sites, the bacterial composition was mainly driven by the sample type (*p* < 0.001) (Figure 2 and Table 2), with a significantly distinct bacterial community among the external sediment, the gut content and the gut tissue samples (Supplementary Table S3). A weaker but significant effect of the sampling site was also observed on the bacterial community composition (Table 2). While this site effect was observed for external sediment and gut content samples (Table 2), it was not detected in the case of the gut tissue samples (Table 2), likely due to the higher variability of bacterial community composition among individual replicates, as visible by the dispersion on the NMDS (Figure 2).

**TABLE 2:** Multivariate permutation test on bacterial community dissimilarities (beta-diversity).

**Sample grouping**	**Df**	**Sums of square**	***F*-statistics**	***R*^2^**	***p*-Value**	**Significance**
Site effect	1	0.578	3.896	0.049	0.002	**
Sample type effect	2	6.013	20.347	0.512	0.001	***
Site*Sample type effect	2	0.733	2.479	0.062	0.009	**
External sediment	1	0.480	26.033	0.813	0.020	*
Gut content	1	0.574	6.480	0.351	0.004	**
Gut membrane	1	0.255	0.937	0.072	0.466	ns

Multivariate permutation test was performed on Bray–curtis dissimilarity matrix of the bacterial community from each sample type and site combination computed. P-values assess the significant effect of the sites and the sample type into the sample grouping. Site effect was additionally evaluated on each sample types. A total of 999 permutations were performed. Significance: ***p-value < 0.001; **p-value < 0.01; *p-value < 0.05; ns, non-significative.

Fifteen OTUs were identified as major drivers of the bacterial community ordination; 4 OTUs of unknown *Gammaproteobacteria*, and the *Flavobacteriaceae* and *Sandaracinaceae* families, 3 OTUs of the *Rhodobacteraceae* and *Pirullulaceae* families, and 8 OTUs of the *Spirochaetaceae*, *Bacteroidetes BD2-2*, *Lentimicrobiaceae* and *Desulfobacteraceae* taxa were discriminant of external sediment, gut content and gut tissue community, respectively (Figure 2, Supplementary Table S3). The closest relative sequences of OTU-2, OTU-7, and OTU-11were previously found in marine sediment from the same area (i.e., King George Island) (Vázquez et al., 2017) (Supplementary Table S4).

### Functional Attribute Predictions of the *A. agassizii* Microbiota

**FIGURE 3:**
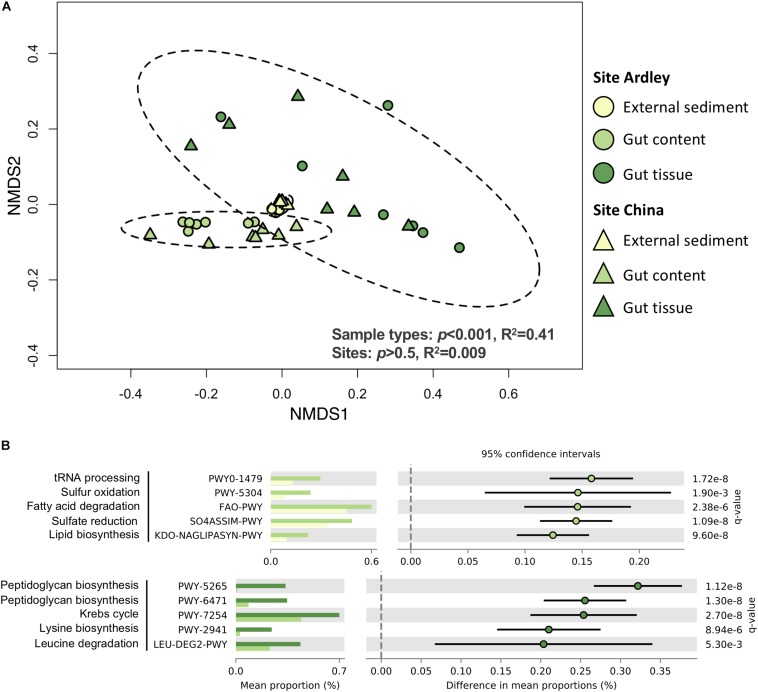
Functional differentiation of the bacterial communities from the *Abatus agassizii* gut microbiota determined using PICRUSt v2.2.0. **(A)** NMDS ordination of the MetaCyc pathways composition of the external sediment and the *Abatus agassizii* gut microbiota. Colors are assigned to the different sample types. Circles and triangles represent samples from Ardley and China sites, respectively. Ellipses represent the standard deviation of all points for each sample type, with a confidence interval at 0.95. Strength and significance of sample groupings according to the sample type and the site were tested using permutational multivariate analysis of variance (Adonis test) on Bray–Curtis distance matrix computed from pathways’ abundance table. **(B)** Details of the top 5 MetaCyc pathways significantly enriched either in the gut tissue or in the gut content. Colors are assigned to the different sample type. Relative abundances and *p*-values obtained from the two-sided Welch’s test corrected with the Benjamini–Hochberg FDR method are provided. Comparisons with *p*-values < 0.05 were considered significant.

A total of 2063 Enzyme Commission (EC) numbers and 409 MetaCyc pathways were identified from the 1015 OTUs of the study. As for bacterial taxonomic composition, the sample type was the major factor driving bacterial functional composition in both EC numbers and MetaCyc pathways, explaining more than 40% of the variance observed (Figure 3A, Supplementary Table S5). Each sample type was different from the two others based on its EC numbers and MetaCyc pathways composition (Supplementary Table S5). Besides, the abundances of EC numbers and MetaCyc pathways significantly decreased from the external sediment to the gut tissue suggesting a gradient of microbiota specialization (Supplementary Figure S2). No site effect was observed on EC numbers nor MetaCyc pathways (*p* > 0.5, *R*^2^ = 0.009, Figure 3, Supplementary Table S5), contrary to the slight site-effect observed at the taxonomic level, which could be explained by functional redundancy. The relative abundance of 1233 ECs and 296 MetaCyc pathways were significantly different among the three sample types (data not shown), suggesting a functional differentiation of the community along the digestive tract. The five bacterial functions most enriched in the gut content in comparison with the external sediment were related to tRNA processing, sulfur metabolism, fatty acid degradation and lipid synthesis (Figure 3B). The five functions most enriched in the gut tissue in comparison with the gut content samples were related to the peptidoglycan synthesis, the Krebs cycle and amino acids synthesis/degradation (Figure 3B).

### Sample Type Specificity of Bacterial OTUs

**FIGURE 4:**
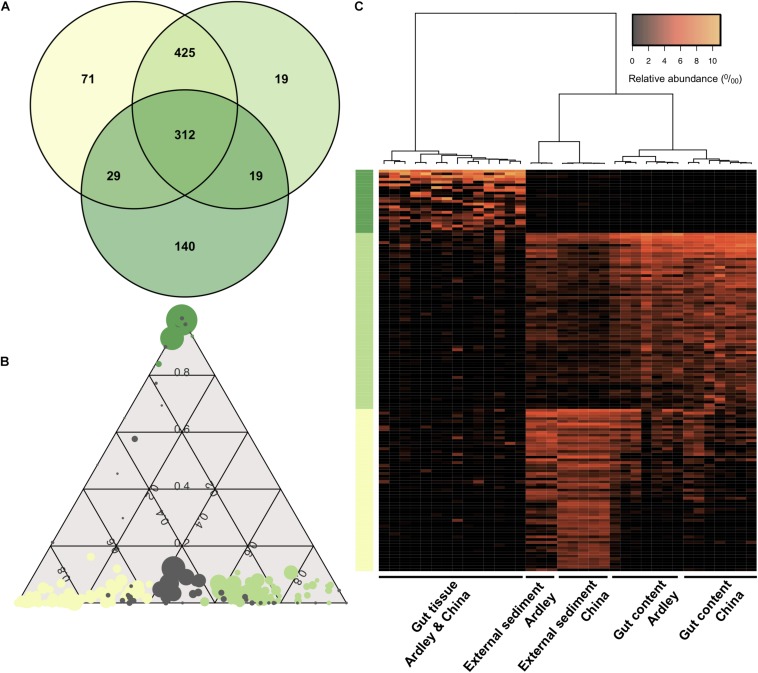
Distinctive sample type specificity of the *Abatus agassizii* microbiota. **(A)** Venn diagram of shared and specific OTUs for the three sample types (based on the total OTU table). The number of OTUs in each biological sample type is shown. Dark green, light green and aniseed yellow represent the external sediment, the gut content and the gut tissue, respectively. **(B)** Ternary plot of OTU compositions. Each circle represents one OTU. The size of each circle represents the relative abundance of the OTU (minimum of 0.5% relative abundance). The position of each circle represents its contribution to the relative total abundance of the biological sample type considered. Dark green, light green and aniseed yellow circles mark OTUs significantly enriched in the gut tissue, in the gut content and in the external sediment, respectively (FDR < 0.05). Gray circles represent OTUs without any significative enrichment. **(C)** Heatmap of the external, gut content and gut tissue enriched OTUs. Vertical columns represent samples. Horizontal rows represent OTUs (minimum of 0.5% relative abundance). Clustering at the top is computed using Pearson correlation coefficient as distance measurement and Ward agglomeration method. Colors on the left side represent OTU sample type enrichment as previously described.

We used a Venn diagram to evaluate the shared versus the exclusive OTUs among sample types. About 30% of the OTUs were shared among the external sediment, the gut content and the gut tissue samples (Figure 4A). The gut tissue presents the highest number of unique OTUs compared with the other sample types (28% of the total gut tissue OTUs), while the external sediment and the gut content shared 84% of their OTUs between each other (Figure 4A). To further identify the bacterial OTUs’ profiles of each sample type that were responsible for the observed differentiation of external sediment and *A. agassizii* microbiota, pairwise comparisons of moderated *t*-tests were used and the identified OTUs were represented under a ternary plot (Figure 4B). From the 837, 775 and 500 distinct OTUs present in the external sediment, the gut content and the gut tissue samples, we found 59, 64, and 23 OTUs significantly enriched in the external sediment, the gut content and the gut tissue, respectively (e.g., more abundant in external sediment versus gut content and in external sediment versus gut tissue, FDR < 0.05) (Figures 4A,B). These OTUs, enriched in the external sediment, the gut content and the gut tissue, were mainly *Flavobacteriaceae* (34%), *Pirellulaceae* (58%), and *Spirochaetaceae* (35%), respectively (Supplementary Figure S3), and were consistent with the previous mentioned 15 OTUs that mostly drive the bacterial community ordination (Section “Sample Type Specificity of Bacterial OTUs,” Figure 2). Based on these OTUs-enriched profiles, all samples clustered according to the sample type (Figure 4C). Consistent with the previous Bray–Curtis analysis and unlike to the gut tissue, the clustering of external sediment OTUs-enriched profiles, and in a lesser extent for gut content, is marked by the effect of the site (Figure 4C).

### Core Microbiota, Co-occurrence Networks and Neutral Model

To identify consistent co-occurrence patterns within the microbial communities between the two *A. agassizii* populations and limit the potential noise linked to the site, we focused on shared taxa between Ardley and China sites. We defined a gut core microbiota of the two sites, composed of 136 and 7 OTUs from the gut content and the gut tissue sample types, respectively (Supplementary Figure S4).

**FIGURE 5:**
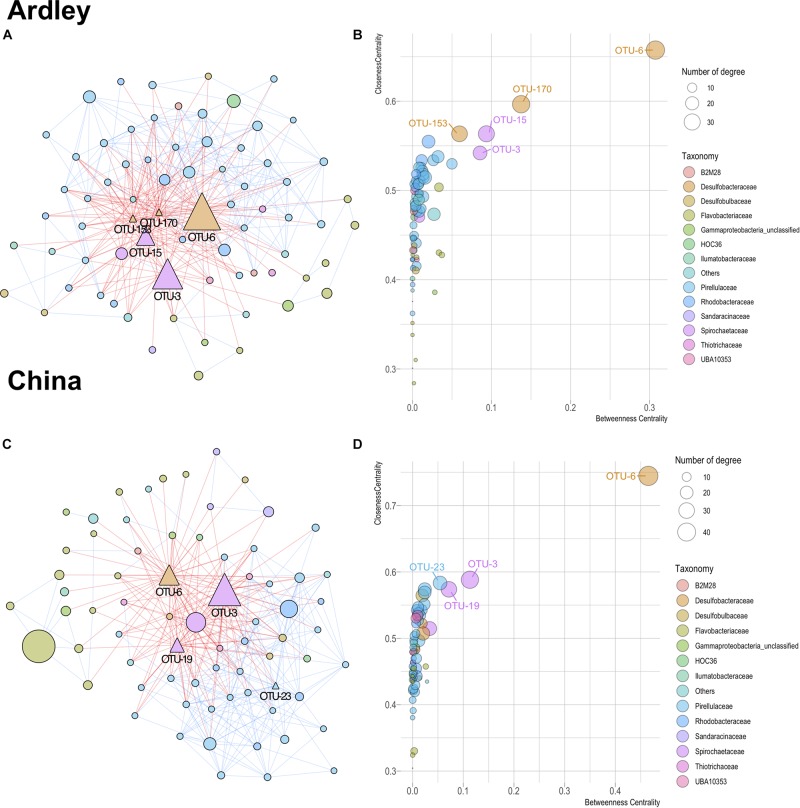
Co-occurrence patterns of bacterial taxa from gut microbiota (content and tissue) of *Abatus agassiizi*, determined through Co-occurrence Network inference (CoNet v1.1.1) and analyzed using Cytoscape (v3.6.0). Panels (**A–D)** refer to the *Abatus* populations of Ardley and China sites, respectively. Networks were built from the 321 OTUs coming from the core China/Ardley only. The networks **(A,C)** were edited using the Compound Spring Embedder algorithm in Cytoscape. Edges are drawn with red and blue lines representing co-exclusion and co-presence interactions, respectively. Nodes represent the bacterial OTUs and were colored according to their taxonomic affiliations (at the family level). Only OTUs with significant edges are represented. Nodes’ size is scaled on the abundance of the OTU (number of reads). Triangles in the networks indicate the hub taxa, defined as nodes with high centrality (i.e., closeness and betweenness) and high connectivity (i.e., node degree) **(B,D)**.

Theoretical relationships among the 143 OTUs of the core gut microbiota (i.e., content and tissue) taxa were computed on each site. Both networks harbored comparable characteristics with 357 edges among 72 OTUs, and 350 edges among 71 OTUs for the Ardley and China populations, respectively (Figure 5). More detailed properties provided by NetworkAnalyzer confirmed the similitude in terms of average network centralization (Ardley 0.421, China 0.478), characteristic path length (Ardley 2.199, China 2.137), average number of neighbors (Ardley 9.917, China 9.859), network density (Ardley 0.140, China 0.141) and heterogeneity (Ardley 0.793, China 0.852). In both networks, the predicted interactions were relatively balanced between co-exclusion and co-presence (182 and 157 negative edges vs 175 and 193 positive edges for Ardley and China, respectively), (Figures 5A,C). Based on their closeness centrality, betweenness centrality and the number of associations in the networks, 5 and 4 OTUs were identified as potential “hub taxa” in *A. agassizzi* microbiota of Ardley and China, respectively (Figures 5B,D). The hub taxa belonged to the *Spirochaetaceae*, *Desulfobacteraceae*, and *Pirellulaceae* families. All of these hub taxa, apart from the OTU-23, mainly belonged to the gut tissue (Supplementary Figure S3), suggesting that the gut tissue community members are the main drivers of the whole gut microbiota composition. The same OTU (OTU-6, *Desulfobacteraceae*) was identified in both populations as the most likely hub taxon and harbored exclusively negative relationships with the other nodes (Figures 5A–D).

**FIGURE 6:**
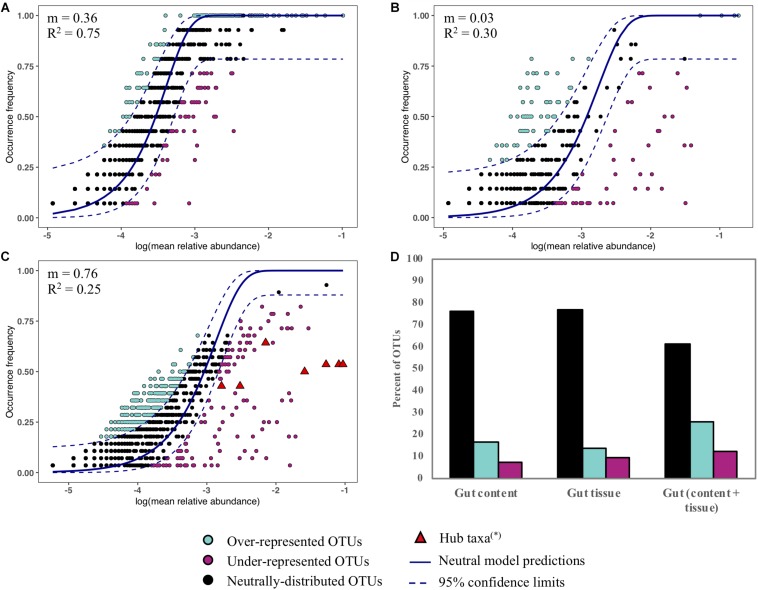
Fit to the neutral model for the gut content **(A)**, the gut tissue **(B)** and the gut microbiota (content and tissue) **(C)**. Each point represents an OTU and the color indicates its fitting to the neutral model. The predicted occurrence frequency and the 95% confidence interval (CI) around the neutral model prediction are drawn. OTUs that are within the confidence limits are well-predicted in terms of frequency and abundance by the neutral model. OTUs that are above the upper CI limit are over-represented in the samples. OTUs that are below the lower CI limit are under-represented in the samples. (^∗^) OTUs previously identified as hub taxa in the co-occurrence analysis are highlighted. **(D)** Percent of OTUs that are within, above and below the 95% confidence limits of the neutral model.

We assessed the contribution of neutral processes in the assembly of *Abatus* gut content, gut tissue and both content and tissue (Figures 6A–C). In all cases, the neutral model outperformed the binomial distribution model, as indicated by the lower AIC values (neutral; −642, −39, −299 vs binomial; −608, 183, −89, for the gut content, the gut tissue and the gut microbiota, respectively). The frequencies and abundances of 61–84% OTUs were well-predicted by the neutral model indicating that stochastic factors mainly shape the *Abatus* microbiota (Figure 6D). The fit of the neutral model and the migration rate *m* strongly decreased in the gut tissue (*m* = 0.03, *R*^2^ = 0.30) compared to the gut content (*m* = 0.36, *R*^2^ = 0.75), suggesting that non-neutral processes such as host selection, microbial interactions or active dispersal increase as the microbiota is more closely related to the host tissue (Figures 6A,B). Similar OTUs proportions (∼23%) of gut content and gut tissue microbiota were above (i.e., over-represented) and below (i.e under-represented) the predictions of the neutral model (Figure 6D). This proportion was twice higher (∼39%) for gut microbiota OTUs (both content and tissue). The hub taxa previously identified through the co-occurrence analysis deviate from the neutral model predictions (i.e., under-represented) in gut microbiota (Figure 6C).

## Discussion

### Abatus Sea Urchins Act as a Selective Filter of the Surrounding Environment Microbiota

In this study, we characterized for the first time the gut microbiota of a deposit-feeder irregular sea urchin living in Antarctica through a metabarcoding approach. Contrastingly with the general pattern observed for terrestrial invertebrate microbiota (Ley et al., 2008), *Abatus* gut communities did not cluster with communities from the surrounding environment. Indeed, we found a marked differentiation of the *A. agassizii* microbiota, at the taxonomic level, with divergent bacterial assemblages among the external sediment, the content and the tissue of the digestive tract. These shifts in bacterial beta-diversity were associated with a significant and progressive decrease of alpha diversity from the external sediment to the gut tissue. These results indicate that the conditions within the *Abatus* host impose successional ecological filters sieving the bacterial diversity of the surrounding sediments and filtering out certain taxa (Stagaman et al., 2017). Analogous differentiation patterns of gut microbiota between gut tissue and digesta have been described in regular sea urchins with a nutrition mode based on a chewing organ (Aristotle’s lantern) allowing them to digest algae (Hakim et al., 2016; Hakim et al., 2019), and in corals (Yao et al., 2019). However, given the feeding-mode of *Abatus* sea urchins and the likeness between their feeding material and their gut content, the detection of distinct bacterial community compositions was unexpected between the external sediment and the gut content samples. This result suggests significant modifications of the environmental conditions within the gut, such as the nutrient availability due to the efficiency of absorption by the host cells, the physicochemical properties, the local pH and oxygen content (Hacquard et al., 2015). Mutualistic, antagonistic and facilitation interactions with other members of the gut microbiota and/or host immune factors may also shape the gut content and the gut tissue bacterial communities differentially (Moran et al., 2019). The transient microbiota, consisting of allochthonous and short-term visitors associated to the gut content, is classically distinguished from the resident microbiota that is supposedly more tightly associated to the host and colonizing the mucosal surface of the gut tissue (Zilber-Rosenberg and Rosenberg, 2008; Hammer et al., 2019). In our results, the most similar bacterial communities were the external sediment and the gut content microbiota, suggesting that the gut content is made of more transient bacteria sourced from the diet. Contrarily, the gut tissue microbiota was the most different from the two other sample types communities, indicating that this community would be resident in *Abatus* gut and more impacted by host-related factors.

In addition to the taxonomic differentiation, our study clearly revealed contrasted functional potentials between the gut content and the gut tissue with differential abundances in various metabolic pathway predictions. Two pathways related to the lysine anabolism and leucine catabolism were significantly over-represented in the *Abatus* gut tissue community. One of the main advantages of hosting a gut microbiota is the improved utilization by the host of suboptimal nutrients (Hammer et al., 2019). This feature has been proposed as particularly important in the case of regular sea urchins that may benefit from the amino acids produced by bacterial metabolism (Lawrence et al., 2013). For instance, Fong and Mann (1980) showed the implication of the gut bacterial community of the sea urchin *Stronglylocentrotus droebachiensis* in the synthesis of two amino acids (leucine and lysine) supplied to the host. More recently, Hakim et al. (2016) predicted, using PICRUSt, an elevated metabolism of amino acids in the gut microbiota of the sea urchin *Lytechinus variegatus* in comparison with the surrounding environment. While our over-represented or over-abundant functional predictions were more related to S-related anaerobic redox processes in the gut content, we found a higher occurrence of Krebs cycle predictions in the gut tissue, supporting the idea that in contrast to the content where anoxic conditions prevail, the gut tissue would be locally more oxygenated (Thorsen, 1998).

### Different Magnitudes of Microbiota Variability Among Sample Types

The Fildes Peninsula in Antarctica remains today a remote and logistically challenging location with few description of its bacterial microbiota, especially of the shallow marine sediments (Foong et al., 2010; Fan et al., 2013). Here, *Gammaproteobacteria* and *Bacteroidetes* were dominant in the external sediment samples of both sites, thus confirming the previous findings in the literature reporting these classes as the most abundant in marine sediments of the Fildes Peninsula in Antarctica (Foong et al., 2010; Fan et al., 2013). It is also worth emphasizing that, despite the important differences in the protocol used (e.g., genomic DNA extraction, PCR primer, among others), we retrieved various OTUs (including the community-structuring ones) strictly identical to those previously identified in the marine benthic sediment of the King George Island (Vázquez et al., 2017), thus validating the robustness of our methodology.

Although ten times lower than the sample type effect, we observed a significant contribution of the sampling site on the bacterial community compositions of external sediments and gut contents (Adonis test, *p* < 0.005, Figure 2). The two sampling locations were relatively close (<2 km) in the same basin (Fildes bay) and are expected to mostly differ by the water depth (i.e., Ardley 7 m and China 2 m). Water depth is a proxy for several physical and chemical variables in the ocean, such as pH and temperature, among others, and has been described as a driving factor of the benthic microbial community (Zinger et al., 2011; Zhu et al., 2013; Liu et al., 2015). Analogously to Gong et al. (2015) who observed a decrease of the microbial diversity with increasing water depth, a trend of a lower richness was observed on Ardley compared with China sampling site. Unlike external sediment and gut content sample types, there was no significant site-effect on the gut tissue communities between individuals from Ardley and China sites, supporting the constraining effect of the host factors on the environmental bacterial diversity. Unexpectedly, the variability within gut tissue samples was nearly twice of that within external sediment samples. The host genotype (e.g immunity) may shape the gut microbial composition by modifying the abundance of specific taxa, overwhelming the idiosyncratic variations linked to the site and leading to the individualization of the microbiota within a same species as observed in other vertebrate and invertebrate models (Benson et al., 2010; Stagaman et al., 2017; Ravenscraft et al., 2019). Furthermore, despite the cautious homogenization procedure aforementioned, we cannot discard the hypothesis that the higher variability among the gut tissue samples might be an artifact of the microbiota spatial partition along the digestive tract. Thorsen (1998) previously reported contrasting values of pH, oxygen and sulfates in distinct regions of the digestive tract of the deposit-feeder *Echninocardium cordatum*. Given the similitudes of the gut (i.e., structure and size) and the sediment-dweller behavior of *Echinocardium* and *Abatus* (Ziegler, 2014), it is reasonable to assume that *Abatus* may also harbor distinct metabolic activities and physicochemical properties according to the gut region, leading to the subsequent partition of its microbiota. Future research on the *Abatus* gut microbiota should consider and evaluate the differentiation of the gut microbiota along the digestive tract.

### Composition Specificity of the Abatus Microbiota

The taxonomic composition of *A. agassizii* gut content microbiotasubstantially diverged from those previously reported in regular urchin species, where *Fusobacteriia* and *Proteobacteria* were dominant (Becker et al., 2009; Yao et al., 2019). The difference of the diet (i.e., mainly grazers and scrapers versus deposit-feeders) may be very likely involved, since it is known to mainly shape the sea urchin gut microbiota (Yao et al., 2019), as most other host-microbiota. In contrast with González-Aravena et al. (2016) who found the microbiota composition of the regular Antarctic sea-urchin *Sterechinus neumayerii* dominated by *Alphaproteobacteria* and *Flavobacteria* and relatively similar to the seawater’s one, we noticed a shift in the dominant class of the gut content microbiota of *A. agassizii* with a marked enrichment in *Plantomycetacia* mostly represented by the *Blastopirellula* genus (20% of the Planctomycetes sequences). The *Planctomycetes* phylum (including the *Planctomycetacia*) has been observed at various abundances in several marine microbiotas, such as sponges (Taylor et al., 2013; Rodriguez-Marconi et al., 2015), tubeworms (Medina-Silva et al., 2018), jellyfish (Lee et al., 2018), macroalgae (Bengtsson and Øvreås, 2010) and regular sea urchins (Hakim et al., 2016). However, such a predominance has never been reported so far in marine invertebrates. Accumulating evidences suggest that *Planctomycetacia* belonging to the *Rhodopirellula*-*Pirellula*-*Blastopirellula* clade could play a major role in the degradation of sulfated polymeric carbon through sulfatase enzyme activity (Glöckner et al., 2003; Wegner et al., 2013), notably under anaerobic conditions (Elshahed et al., 2007), and in interaction with marine macro-organisms (Hempel et al., 2008; Bengtsson and Øvreås, 2010; Medina-Silva et al., 2018). Therefore, a reasonable explanation for the enrichment of *Planctomycetacia* in the *Abatus* gut content would be its high concentration in sulfated compounds coupled with a low oxygenation rate, conditions that are known to occur in other irregular sea urchins (Plante and Jumars, 1992; Thorsen, 1998). These sulphated compounds, found in marine photosynthetic organisms like microalgae and seaweeds (Bengtsson and Øvreås, 2010), may be deposited on the seafloor at organisms’ death and ingested by the *Abatus* host together with the sediment. Intestinal secretions from the echinoid host itself are also a substantial source of sulphated mucopolysaccharides (Da Silva et al., 2006). Consistently, we predicted through PICRUSt an elevated sulfur metabolism in the *Abatus* gut content (i.e., sulfur oxidation and sulfate reduction) probably explained to some extent by the *Planctomycetacia* enrichment. An active metabolism of sulfur has been suggested in *E. cordatum* gut, supported by the substantial sulfate and sulfide concentrations resulting from sulfatase enzymes and sulfate-reducing bacteria activity (Thorsen, 1998; Thorsen et al., 2003).

### Spirochaeta and Desulfobacula: Keystones of the Abatus Microbiota

We defined a core gut microbiota was defined for gut contents and gut tissues between the two populations of *A. agassizii* (China and Ardley sites). Given the expected tight interactions between transient and resident microbial communities, we explored the relationships among taxa between the gut content and the gut tissue, according to their significant patterns of co-occurrence and co-exclusion (Faust et al., 2012). Based on their high connectivity in the network of each site, OTUs from two families, *Spirochaetaceae* and *Desulfobacteraceae*, were identified as hub taxa of *A. agassizii* gut microbiota and could be potential keystones occupying a crucial functional role in the host physiology (Berry and Widder, 2014; Banerjee et al., 2018).

The gut tissue of *A. agassizii* was dominated by *Spirochaetia*, mostly represented by the genus *Spirochaeta* which was prevalent in all of the samples and accounted for as much as 80% relative abundance of the class. *Spirochaetes* are motile free-living, facultative/obligate anaerobes (Leschine et al., 2006), associated with numerous marine invertebrates, among others, corals (Lawler et al., 2016; Van De Water et al., 2016), sponges (Matcher et al., 2017; Kellogg, 2019), sea stars (Jackson et al., 2018), and at the body surface of the regular sea urchin species *Tripneustes gratilla* (<15% relative abundance) (Brink et al., 2019). Such a prevalence inside the gut of a sea urchin has never been described and suggests a specific interaction between *Spirochaeta* and the *Abatus* host. Interestingly, nitrogen fixation activity was reported in several free-living strains of *Spirochaeta*. Further, the *nifH* gene of these strains was phylogenetically close to the *nifH* sequences of clones encountered in Antarctica (Lilburn et al., 2001). Moreover, Guerinot and Patriquin (1981) reported bacterial nitrogen fixation activity in several regular sea urchin species from temperate, tropical and arctic seas. Thus, it is tempting to expect a spirochete-specific N_2_ fixation metabolism inside the gut that might benefit *A. agassizii* by increasing its nitrogen supply in a context of low-nitrogen food. Direct quantification of the functional gene *nifH* or nitrogenase activity measurement performed on the gut could confirm this N_2_ fixation metabolism in the future.

Remarkably, the keystone top candidate of the two *Abatus* populations was the very same OTU from *Desulfobacteraceae* (OTU-6 close to the *Desulfobacula* genus). Taxa belonging to the *Desulfobacteraceae* family have been previously identified in the literature as potential keystone in other regular (Meziti et al., 2007; Hakim et al., 2016; Hakim et al., 2019) and irregular sea urchin species (Thorsen et al., 2003; Da Silva et al., 2006). The *Desulfobacteraceae* family members (including *Desulfobacula*) are sulfate-reducers known to be quantitatively important in the anaerobic degradation of organic matter in coastal marine sediments (Jørgensen, 1982; Bowles et al., 2014). Thus, their prevalence and enrichment in the gut could be directly linked to the deposit-feeding mode of *A. agassizii*. Furthermore, these sulfate-reducing bacteria hosted on the *Abatus* gut tissue may take advantage of the sulfate enrichment in the lumen, that derive from the fermentative degradation of complex sulphated polysaccharides achieved by the gut content microbiota (see the previous section) (Thorsen et al., 2003; Da Silva et al., 2006). In return, the host may benefit from the bacterial exudation and from the bacterial degradation of the recalcitrant organic compounds present in the sediment. Likewise, some strains would also be able to directly contribute to the anaerobic degradation of complex aromatic compounds, as previously documented in the *Desulfobacula* genus (Raber et al., 1998; Kim et al., 2014).

The reduction of sulfated substrates is ineluctably associated with the accumulation of sulfides potentially toxic for both host and microbiota (De Ridder et al., 1985; Vismann, 1991; Thorsen et al., 2003). The sulfide toxicity could explain that all interactions implying the OTU-6 with the other core taxa were co-exclusions, at the exception of *Spirochaeta* keystone OTUs. Previous studies have described several *Spirochaeta* strains as resistant to classically deleterious sulfide concentrations and also able to oxidize sulfides under aerobic conditions, as the ones present at the proximity of the host intestinal tissue (Hoover et al., 2003; Dubinina et al., 2011; Miyazaki et al., 2014). Thus, *Spirochaeta* may endorse the role of detoxifiers in the *Abatus* gut preventing the sulfide accumulation by oxidizing it into elemental sulfur. The syntrophic relationship between sulfide oxidizers and sulfate reducers has been described accordingly in sediment-dweller gutless worms (Blazejak et al., 2005), and aggregation of *Desulfonema* (genus of *Desulfobacteraceae*) and *Spirochaeta* has been observed in culture (Da Silva et al., 2006).

### Abatus Sea Urchins Have a Unique Resident Microbiota

The application of a neutral model to *A. agassizii* microbiota data has laid the first stones into the comprehension of keystone taxa assembly in the gut of *Abatus*. We showed that the gut tissue microbiota fits less to the neutral model than the gut content microbiota, thus indicating that the gut tissue microbiota would be less influenced by stochastic factors (i.e., ecological drift and passive dispersal) than the gut content community. This result underlines the fact that, the tighter the association between the microbiota and *Abatus* gut tissue is, the stronger deterministic factors (e.g., host factors) may shape the gut bacterial taxa abundance and frequency. As the acquisition of mutualistic bacteria by marine invertebrates generally occur horizontally (Nyholm and McFall-Ngai, 2004; Dubilier et al., 2008), the most parsimonious mechanism would be a constant inoculation from the bulk environment through the sediment swallowing (i.e., horizontal acquisition), carrying opportunistic bacteria that fit the very selective conditions of *Abatus* gut tissue. Alternatively, the sea urchins may also acquire their microbiome from the environment at some early point in their development, though as the gut develops, it creates a highly selective environment (Thorsen, 1998) that enriches in these microbes and paves the way to the remarkable difference observed in the gut microbial community compared to the sediment (external and contained in the gut). A recent study identified both horizontal and vertical acquisition mechanisms as explaining factors of the symbiont genetic diversity in the brooding coral species *Seriatopora hystrix* (Quigley et al., 2018). In the case of brooding sea urchin species like *A. agassizii*, the inheritance by the juveniles of a part adult microbiota and the mechanisms responsible to the keystone assembly on the gut tissue, remains to be further investigated.

Interestingly, the inspection of the neutral model fit for gut microbiota revealed that the keystone taxa previously identified as prevalents, substantially enriched, and importants in the gut microbiota structure, were also markedly deviated from the predictions of a neutral model. The under-representation pattern observed suggests that these taxa would be to some extent either actively selected against the host or especially dispersal limited (Burns et al., 2016), supporting our rationale that these keystone taxa are well adapted to the gut tissue and are somehow actively driven by the *Abatus* host (Adair et al., 2018). Thus, the keystone taxa assembly in the gut tissue may be the result of an integration by the host, which actively promotes mutualistic bacteria by producing bioactive metabolites (Dubilier et al., 2008; Mangano et al., 2009). Overall, it might be advantageous for the host to maintain stable, functional associations with taxa from its gut microbial community as long as they confer fitness benefits (e.g., improved nutrition in the oligotrophic environment, gut detoxification, among others) (Giudice et al., 2019). A possible syntrophy between *Spirochaeta* and *Desulfobacula* genera, contributing to the organic matter digestion, the N supply, the S cycling and the gut detoxification of *Abatus*, could be actively selected and maintained across *Abatus* individuals and populations.

## Conclusion

In summary, this case study provides a comprehensive description of the microbiota of *A. agassizii*, through its taxonomic composition and predicted metabolic role. We highlighted specific enrichment in bacterial taxa and putative associated functions between the content and the tissue of the gut, congruent with the sediment-dwelling ecology of the host. We revealed the existence of a core microbiota shared between distinct *A. agassizii* populations in King George Island, Antarctica, and we demonstrated its consistency at a local scale with a structuration around two main keystone bacterial taxa that may have a relevant biological role in the host fitness. Our results also suggest a putative active and host-mediated selection of these gut adherent keystones, that represent promising targets for future interspecific studies of the gut microbiota among the *Abatus* genus species. Altogether, these results highly suggest that despite its relative elementary feeding behavior, *Abatus agassizii* may rely on a selective and unique resident microbiota to survive in the marine benthic sediment.

## Data Availability Statement

The datasets generated for this study can be found in the National Center for Biotechnology Information (NCBI) repository, under the accession number PRJNA590493.

## Author Contributions

LC, JO, and EP designed the research. LC and EP performed the sampling and the fieldwork. GS conducted all the laboratory experiments, managed data mining and all analysis, and wrote the manuscript draft. GS, LC, JO, and EP interpreted the data. All authors edited and approved the manuscript.

## Conflict of Interest

The authors declare that the research was conducted in the absence of any commercial or financial relationships that could be construed as a potential conflict of interest.

## Footnotes

1 www.ncbi.nlm.nih.gov
