# Supplementary material for: Characterization of the Gut Microbiota of the Antarctic Heart Urchin (Spatangoida) Abatus agassizii
Source: Front Microbiol. 2020 Feb 28;11:308. doi: 10.3389/fmicb.2020.00308 (PMC7058685; doi:10.3389/fmicb.2020.00308)
Supplement: Supplementary file 1 [file Data_Sheet_1.PDF]

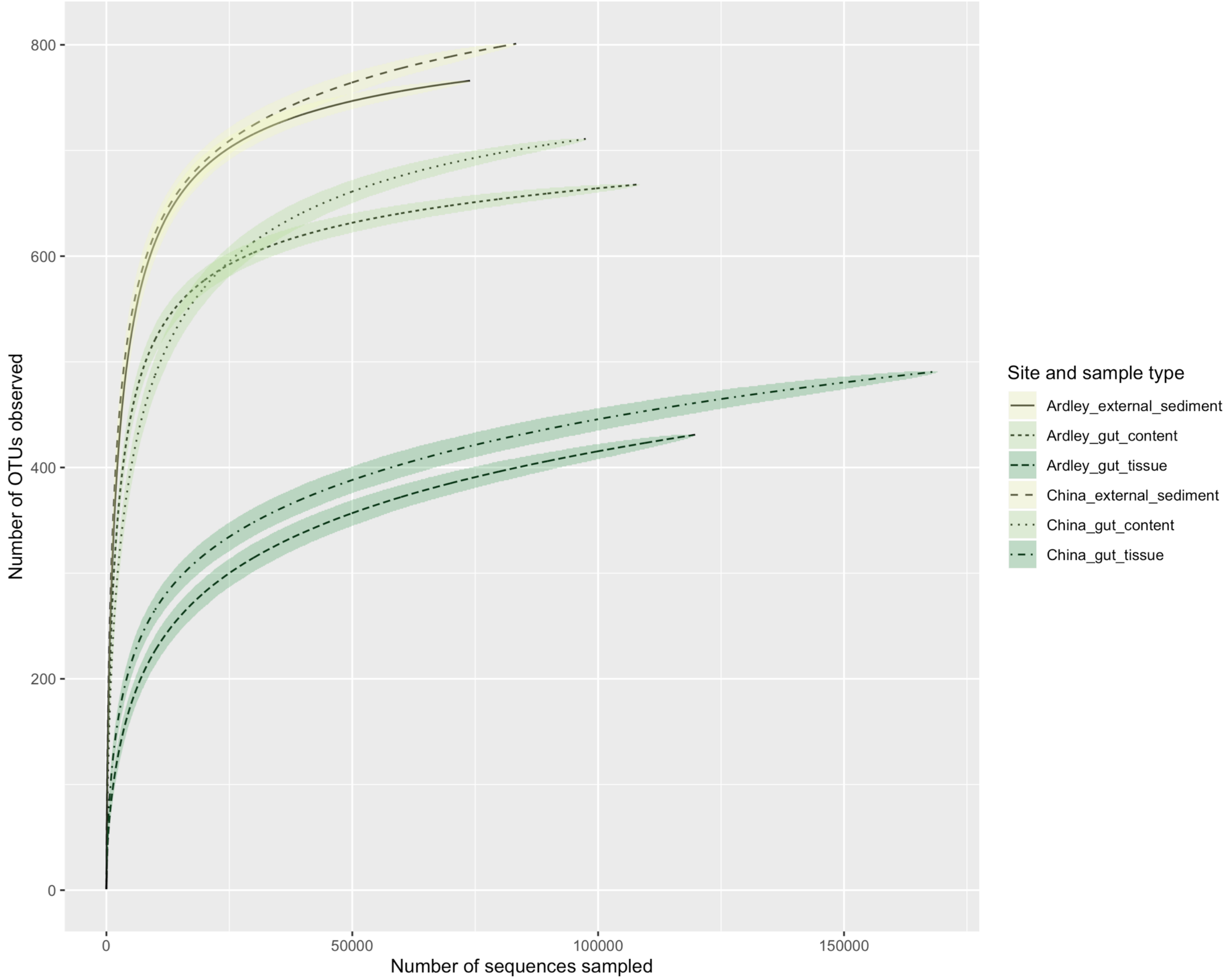

Figure S1. Rarefaction curves of *Abatus agassizii* gut and external sediment samples pooled according to the type and the site of provenance. Linetypes correspond to the sites and sample types combination. Threshold of 0.03 difference was used for the OTU clustering. Confidence intervals at 95% of the observed richness calculated on 5000 sampling iterations are represented. Colours are assigned to the different sample type, aniseed yellow, external sediment; light green, gut content; dark green, gut tissue).

Table S1. Dunn post-hoc tests on alpha diversity indices

Site	Indices	External sediment vs. Gut content	Gut content vs. Gut tissue	External sediment vs. Gut tissue
Ardley	Richness	1.43 / 0.076	2.59 / 0.010*	3.44 / 0.001*
	Shannon	1.43 / 0.076	2.59 / 0.010*	3.44 / 0.000*
	Simpson	1.43 / 0.076	2.59 / 0.010*	3.44 / 0.000*
China	Richness	1.82 / 0.034*	2.33 / 0.020*	3.95 / 0.000*
	Shannon	1.82 / 0.034*	2.33 / 0.020*	3.95 / 0.000*
	Simpson	1.82 / 0.034*	2.33 / 0.020*	3.95 / 0.000*
Sites pooled	Richness	2.36 / 0.010*	3.52 / 0.000*	5.35 / 0.000*
	Shannon	2.36 / 0.010*	3.52 / 0.000*	5.35 / 0.000*
	Simpson	2.27 / 0.012*	3.55 / 0.000*	5.30 / 0.000*

Dunn z test stastistics / *p* -values are provided. * indicate significance of the *p* -values. ($p < 0.05$).

Table S2. ***P-*** **values of wilcoxon pairwise comparisons of bacterial composition at class level among sample types and sites.**

	Ardley			China			Ardley versus China		
**Bacterial class**	**Ext - Con**	**Con - Tis**	**Ext - Tis**	**Ext - Con**	**Con - Tis**	**Ext - Tis**	**Ext A - Ext C**	**Con A - Con C**	**Tis A - Tis C**
Bacteroidia	**0.050**	0.380	0.370	0.100	0.259	0.100	**0.030**	**0.050**	1.000
Spirochaetia	0.090	**0.004**	**0.030**	0.060	**0.005**	**0.010**	0.650	0.530	0.800
Planctomycetacia	**0.030**	**0.002**	**0.030**	**0.005**	**0.002**	**0.005**	**0.030**	0.260	0.080
Gammaproteobacteria	**0.030**	**0.002**	**0.030**	**0.008**	**0.050**	**0.050**	**0.040**	**0.050**	0.710
Deltaproteobacteria	1.000	0.220	0.230	**0.020**	1.000	1.000	0.570	0.700	0.260
Alphaproteobacteria	**0.030**	**0.002**	**0.030**	**0.008**	**0.010**	**0.050**	0.180	0.800	0.100
Latescibacteria_cl	0.060	0.480	0.820	**0.020**	**0.040**	0.640	0.650	0.560	0.370
Acidimicrobiia	**0.030**	**0.006**	**0.030**	**0.01**	**0.006**	**0.010**	0.180	**0.002**	0.360
Thermoanaerobaculia	0.070	0.070	0.070	0.430	**0.010**	0.010	0.140	0.460	1.000
Kiritimatiellae	0.250	1.000	1.000	0.930	0.670	0.670	0.390	0.020	0.940
Clostridia	0.760	0.950	0.760	0.650	0.900	0.650	**0.030**	0.080	0.440

Ext; External sediment, Con; gut content, Tis; gut tissue. The wilcoxon test has been used to perform pairwise comparisons on bacterial composition at class level among the sample types for each site and for each sample type between the two sites. Corrected *p* -values with the Holm's method are shown. Comparisons with *p* -values < 0.05 were considered significant.

Table S3. Pairwise adonis on bacterial community dissimilarities among sample types (beta-diversity)

Site	Sample grouping	Df	Sums of square	F-statistics	R^2	*p* -value	*p* -value adjusted	Significance
Ardley	External sediment vs Gut content	1	0.692	8.866	0.526	0.009	0.027	*
	Gut content vs Gut tissue	1	2.227	11.433	0.488	0.001	0.003	**
	External sediment vs Gut tissue	1	1.465	6.478	0.447	0.009	0.027	*
China	External sediment vs Gut content	1	0.992	18.101	0.644	0.002	0.006	**
	Gut content vs Gut tissue	1	2.260	13.660	0.532	0.001	0.003	**
	External sediment vs Gut tissue	1	2.019	12.942	0.564	0.002	0.006	**

Significance: **, *p* -value < 0.01, *, *p* -value < 0.05

Table S4. Details of the OTUs identified as major drivers of the bacterial community ordination

OTU ID	Tax. Assignation SILVA	Closest relative	Identity (%)	Location	References
OTU-1	Flavobacteriaceae (Fl)	Uncultured bacterium clone G6 T2d	100	Marine sediment offshore of Brest, France	Stauffert *et al.* 2013
OTU-2	Rhodobacteraceae (Rh)	Uncultured bacterium clone S1B1S_11-075	100	Marine sediment from Potter Cove, King George Island, Antartica	Vazquez *et al.* 2017
OTU-3	Spirochaetaceae (Sp)	Uncultured bacterium clone s110	97.07	Marine sediment from the Great Sippewisset salt marsh on Cape Cod, Mass	Stoeck *et al* . 2003
OTU-4	Pirellulaceae (Pi)	Uncultured bacterium clone V1B07b6	100	White microbial film on pebble substrate in the aphotic zone of a shallow hydrothermal vent, South Tonga Arc	Murdock *et al.* 2010
OTU-6	Desulfobacteraceae (De)	Uncultured bacterium clone New_Zealand_cold_seep_clone_NZ_309_Bac24	98.41	Deep sea sediments from Hikurango continental margin, New Zealand	Ruff *et al.* 2013
OTU-7	Pirellulaceae (Pi)	Uncultured bacterium clone S1B1S_10-093	100	Marine sediment from Potter Cove, King George Island, Antartica	Vázquez *et al.* 2017
OTU-9	Gammaproteobacteria unclassified (Ga)	Uncultured bacterium clone ExkYyy31	99.73	Gulf of Mexico sediment	Yang *et al.* 2016
OTU-11	Sandaracinaceae (Sa)	Uncultured bacterium clone S1B1S_10-083	100	Marine sediment from Potter Cove, King George Island, Antartica	Vazquez *et al.* 2017
OTU-15	Spirochaetaceae (Sp)	Uncultured bacterium clone AMSMV-0-B99	98.89	Amsterdam mud volcano sediment, East Mediterranean Sea	Pachiadaki *et al.* 2011
OTU-19	Spirochaetaceae (Sp)	Uncultured bacterium clone s110	96.53	Marine sediment from the Great Sippewisset salt marsh on Cape Cod, Mass	Stoeck *et al* . 2003
OTU-26	Flavobacteriaceae (Fl)	Uncultured bacterium clone LS_3433_bac_A3	99.73	Marine sediment from the Hydrate Ridge North offshore of Oregon, USA	Marlow *et al.* 2014
OTU-43	Spirochaetaceae (Sp)	Uncultured bacterium clone s110 16S	96.80	Marine sediment from the Great Sippewisset salt marsh on Cape Cod, Mass	Stoeck *et al* . 2003
OTU-58	Bacteroidetes BD2-2 (Ba)	Uncultured bacterium clone C5 NEREIS-BAL T270d	99.00	Marine sediment from Brest, France	Stauffert *et al.* 2013
OTU-59	Lentimicrobiaceae (Le)	Uncultured bacteroidetes bacterium	99.00	Marine sediment beneath area of shellfish aquaculture, Sanriku coast, Japan	Asami *et al.* 2005
OTU-117	Bacteroidetes BD2-2 (Ba)	Uncultured bacterium clone 490CT10B53	99.46	Deep sea sediments from Congo shores	Pozzato *et al.* 2017

The letters indicate the taxonomic identification at the family level of the discriminant OTUs (Ba: Bacteroidetes BD2-2, De: Desulfobacteraceae, Fl: Flavobacteriaceae, Ga: Gammaproteobacteria unclassified, Le: Lentimicrobiaceae, Pi: Pirullulaceae, Rh: Rhodobacteraceae, Sa: Sandaracinaceae, Sp: Spirochaetaceae).

Table S5. Multivariate permutation test on the ECs and pathways' abundance tables of *Abatus agassizii* gut and external sediment bacterial communities.

Sample grouping / Prediction hierarchy	ECs	MetaCyc pathways
Site	0.008	0.009
Sample type	0.403 ***	0.414 ***
Site*Sample type	0.080 *	0.073
External sediment vs. Gut content	0.377 **	0.388 **
Gut content vs. Gut tissue	0.361 **	0.340 **
Gut tissue vs. External sediment	0.209 *	0.211 *

Multivariate permutation test (adonis) was performed on Bray-Curtis dissimilarity matrice of the ECs and MetaCyc pathway abundance tables obtained through the PICRUSt predictions matrix. *P*-values assess the significant effect of the sites and the sample types into the sample grouping. A total of 999 permutations were performed. R2 are provided in the table. Significance: ***, p-value < 0.001, **, p-value < 0.01, *, p-value < 0.05

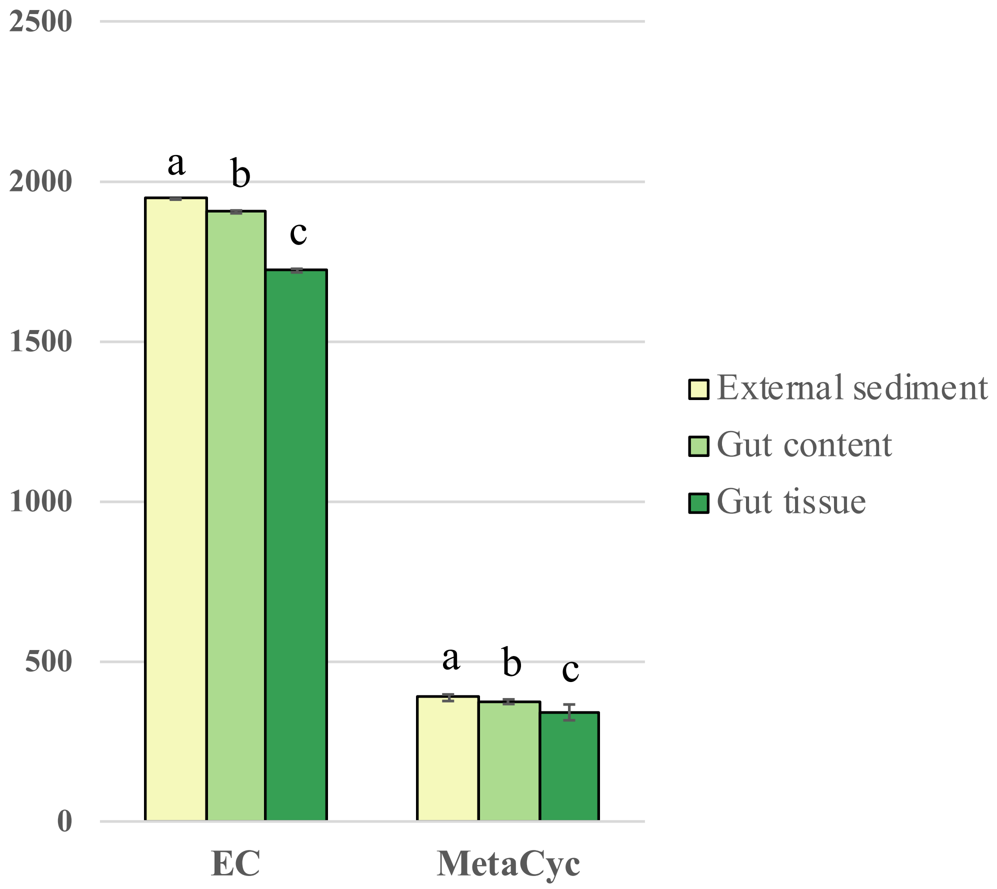

Figure S2. Abundances of EC numbers and MetaCyc pathways according to the sample types. Colours are assigned to the different sample types. Error bars represent the standard error. Abundances were compared using pairwise Wilcoxon tests. Significance threshold was set at p<0.05. Letters indicate significant differences in either EC numbers or MetaCyc pathways' abundance among sample types.

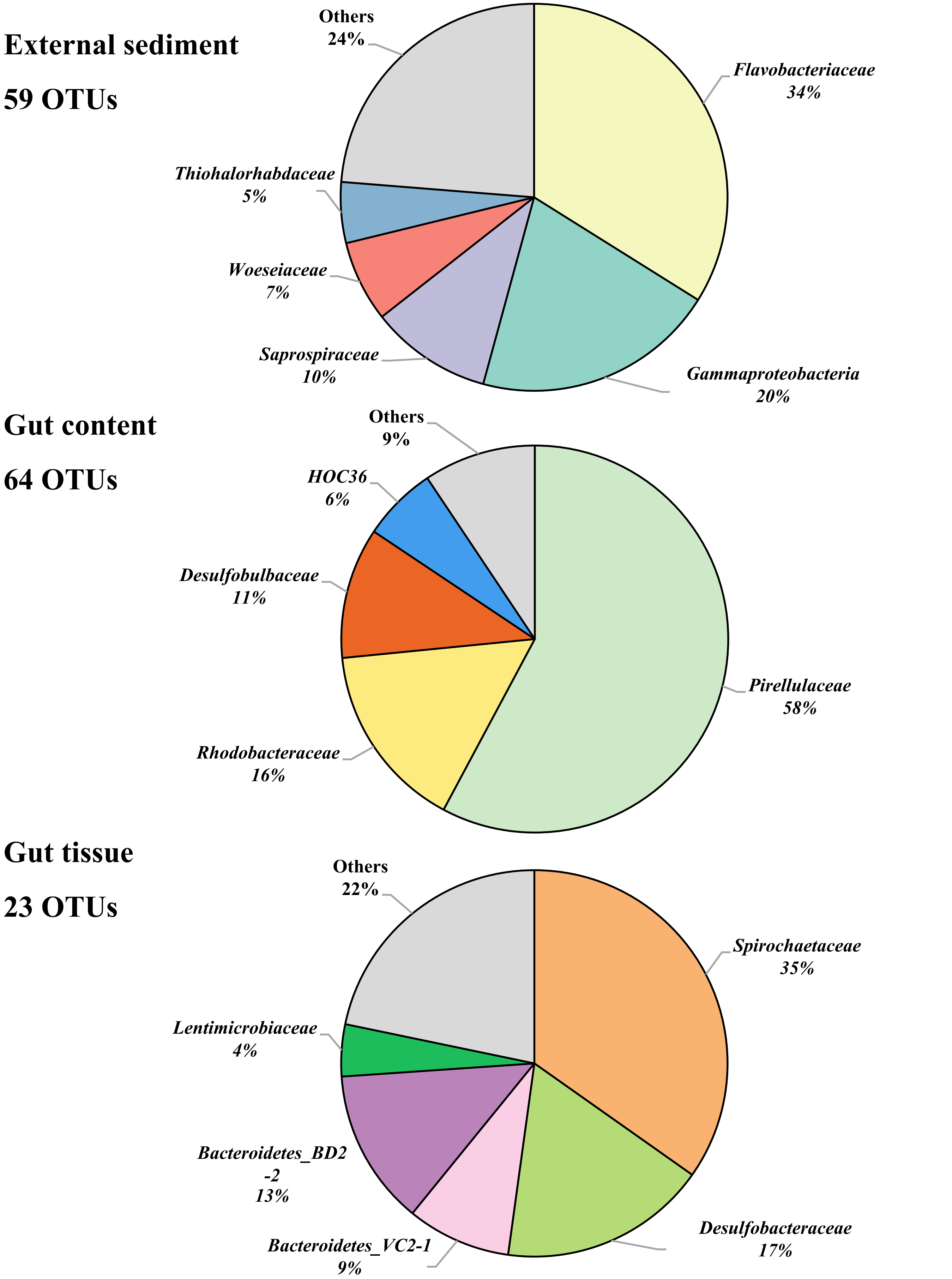

Figure S3. Taxonomic assignations of the enriched 59, 64 and 23 OTUs in the external sediment, the gut content and the gut tissue, respectively.

Assignations are provided at the family level or at the most resolutive scale when family was undetermined. The percentages correspond to the relative abundance of the taxa among the enriched OTUs within a given sample type.

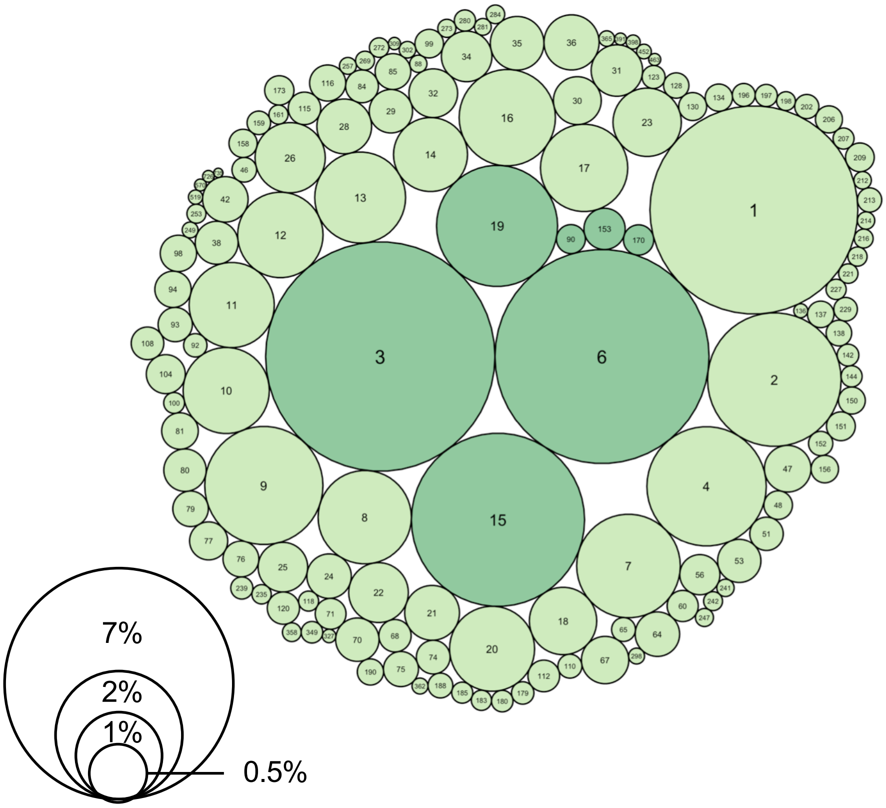

Figure S4. Circle packing representation of the core microbiota OTUs defined at ≥75% prevalence in a same type of samples from both Ardley and China sites.

A total of 136 and 7 OTUs were selected as core microbiota for gut content and gut tissue sample types, respectively. Each circle represents an OTU and its size is scaled on OTU abundance (% of total core sequences). Light green and dark green represent the gut content and the gut tissue, respectively. Numbers correspond to OTU designation. Some OTUs occur in a single or both sample types.

References:

Asami, H., Aida, M., and Watanabe, K. (2005). Accelerated sulfur cycle in coastal marine sediment beneath areas of intensive shellfish aquaculture. *Appl. Environ. Microbiol.* 71**,** 2925-2933.

Marlow, J.J., Steele, J.A., Case, D.H., Connon, S.A., Levin, L.A., and Orphan, V.J. (2014). Microbial abundance and diversity patterns associated with sediments and carbonates from the methane seep environments of Hydrate Ridge, OR. *Frontiers in Marine Science* 1**,** 44.

Murdock, S., Johnson, H., Forget, N., and Juniper, S.K. (2010). Composition and diversity of microbial mats at shallow hydrothermal vents on Volcano 1, South Tonga Arc. *Cahiers de Biologie Marine* 51**,** 407-413.

Pachiadaki, M.G., Kallionaki, A., Dählmann, A., De Lange, G.J., and Kormas, K.A. (2011). Diversity and spatial distribution of prokaryotic communities along a sediment vertical profile of a deep-sea mud volcano. *Microbial ecology* 62**,** 655-668.

Pozzato, L., Cathalot, C., Berrached, C., Toussaint, F., Stetten, E., Caprais, J.-C., Pastor, L., Olu, K., and Rabouille, C. (2017). Early diagenesis in the Congo deep-sea fan sediments dominated by massive terrigenous deposits: Part I–Oxygen consumption and organic carbon mineralization using a micro-electrode approach. *Deep Sea Research Part II: Topical Studies in Oceanography* 142**,** 125-138.

Ruff, S.E., Arnds, J., Knittel, K., Amann, R., Wegener, G., Ramette, A., and Boetius, A. (2013). Microbial communities of deep-sea methane seeps at Hikurangi continental margin (New Zealand). *PLoS One* 8**,** e72627.

Stauffert, M., Cravo-Laureau, C., Jezequel, R., Barantal, S., Cuny, P., Gilbert, F., Cagnon, C., Militon, C., Amouroux, D., and Mahdaoui, F. (2013). Impact of oil on bacterial community structure in bioturbated sediments. *PLoS One* 8**,** e65347.

Stoeck, T., and Epstein, S. (2003). Novel eukaryotic lineages inferred from small-subunit rRNA analyses of oxygen-depleted marine environments. *Appl. Environ. Microbiol.* 69**,** 2657-2663.

Vázquez, S., Monien, P., Pepino Minetti, R., Jurgens, J., Curtosi, A., Villalba Primitz, J., Frickenhaus, S., Abele, D., Mac Cormack, W., and Helmke, E. (2017). Bacterial communities and chemical parameters in soils and coastal sediments in response to diesel spills at Carlini Station, Antarctica. *Sci Total Environ* 605-606**,** 26-37.

Yang, T., Nigro, L.M., Gutierrez, T., Joye, S.B., Highsmith, R., and Teske, A. (2016). Pulsed blooms and persistent oil-degrading bacterial populations in the water column during and after the Deepwater Horizon blowout. *Deep Sea Research Part II: Topical Studies in Oceanography* 129**,** 282-291.
